# The correct name in *Oenothera* for *Gaura drummondii* (Onagraceae)

**DOI:** 10.3897/phytokeys.50.4886

**Published:** 2015-05-27

**Authors:** Warren L. Wagner, Peter C. Hoch, James L. Zarucchi

**Affiliations:** 1 Department of Botany, MRC-166, National Museum of Natural History, Smithsonian Institution, P.O. Box 37012, Washington, DC 20013-7012, USA; 2 Missouri Botanical Garden, P. O. Box 299, St. Louis, Missouri 63166-0299

**Keywords:** *Gaura drummondii*, *Gaura hispida*, *Oenothera hispida*, *Oenothera xenogaura*, nomenclature

## Abstract

In 2007, Wagner and Hoch proposed the new name *Oenothera xenogaura* W.L.Wagner & Hoch for the species then known as *Gaura drummondii* (Spach) Torrey & A. Gray (non *Oenothera drummondii* Hooker, 1834). However, the authors overlooked the availability of *Gaura hispida* Bentham (1840) for this species. Accordingly, we herewith make the appropriate new combination for this species, *Oenothera hispida* (Bentham) W.L.Wagner, Hoch & Zarucchi, and place *Oenothera xenogaura* in synonymy.

## Introduction

The only member of Oenothera sect. Gaura subsect. Xenogaura is a distinctive allopolyploid species that occurs from eastern Texas south through Mexico as far south as Oaxaca. When the genus *Gaura* L. is recognized, the correct name for this species is *Gaura drummondii* (Spach) Torrey & A. Gray, which was used in the revision of the group by Raven and Gregory (1972). Since that time, molecular studies (Hoggard et al. 2004; Levin et al. 2004; Ford and Gottlieb 2007) have shown that *Oenothera* is strongly supported as monophyletic only with the inclusion of *Calylophus* Spach, *Gaura*, and *Stenosiphon* Spach. These four groups also have in common a stigma that either is peltate to discoid, or is deeply to shallowly 4-lobed and then subtended by a more or less conspicuous peltate indusium. These data led Wagner et al. (2007) to broaden the concept of *Oenothera* by including within it *Calylophus*, *Gaura*, and *Stenosiphon*. The new name *Oenothera xenogaura* W.L.Wagner & Hoch was proposed in 2007 for this species when *Gaura drummondii* was transferred to *Oenothera* because use of *Gaura drummondii* is blocked by *Oenothera drummondii* Hooker of sect. *Oenothera*. Within the protologue of their new combination, Wagner and Hoch did not cite any other taxonomic synonym. However, at that time, they failed to take into account *Gaura hispida* Bentham (1840), one of the synonyms included for *Gaura drummondii* by Raven and Gregory (1972). Thus, they missed the opportunity of transferring *Gaura hispida* to *Oenothera* and making the new combination. We herewith correct the mistake and make the appropriate new combination namely *Oenothera hispida* W.L.Wagner, Hoch & Zarucchi. Additionally, along with other synonyms cited by Raven and Gregory, *Oenothera xenogaura*, a legitimate replacement name, is here placed into synonymy. Since Wagner and Hoch did not cite any taxonomic synonym, their *Oenothera xenogaura* was not superfluous when published [Art. 52.1; McNeill et al. 2012].

## Taxonomic part

### Oenothera hispida (Bentham)

Taxon classificationPlantaeMyrtalesOnagraceae

W.L.Wagner, Hoch & Zarucchi comb. nov.

urn:lsid:ipni.org:names:77147316-1

Fig. 1

#### Basionym.

*Gaura hispida* Bentham, Pl. Hartw. 288. 1840.

#### Type.

**Type.** Mexico: In fields near Leon, Guanajuato, June 1837, *Thomas Hartweg 1603* (Holotype: K! [Kew image]; Isotypes: BM, CAMB, G, LD!).

*Schizocarya drummondii* Spach, Nouv. Ann. Mus.Hist. Nat. 4: 382. 1836 [“1835”]. *Gaura drummondii* (Spach) Torrey & A. Gray, Fl. N. Amer. 1: 519. 1838. *Oenothera xenogaura* W.L.Wagner & Hoch, Syst. Bot. Monogr. 83: 213. 2007.

**Type.** U.S.A. Texas: Travis Co., Austin, 1833–1834, *T. Drummond III.36* (Holotype: G; isotypes: BM!, GH!, NY!, P). Note: the BM isotype is mounted on a sheet with two non-type collections of the same species: Purpus 3387 and Purpus 5383.

*Gaura roemeriana* Scheele, Linnaea 21: 579. 1848.

**Type.** U.S.A. Texas: Comal Co., New Braunfels, 1846, *Ferdinand Roemer s.n.* (Lectotype: MO-1833107!, here designated; Isolectotypes: CAS, HAL). The holotype at B was destroyed in World War II.

*Schizocarya crispa* Spach, Nouv. Ann. Mus. Paris 4: 384. 1835. *Gaura crispa* (Spach) D.Dietr., Syn. PI. 2: 1298. 1840.

**Type.** Mexico: Tamaulipas, Matamoros, April 1831, *J. L. Berlandier 2313* (Holotype: G; Isotypes: BM, K, P).

Plant rhizomatous, perennial, forming extensive colonies, strigillose and often also villous. Stems 20–60(-120) cm tall, sometimes strict with a single unbranched main stem but usually somewhat decumbent with several branches from the base and usually irregular branching above. Leaves in a basal rosette and cauline, 0.5–7.5 (-9.5) × 0.1–2.2 cm; subsessile; blade narrowly lanceolate to elliptic, margin subentire to shallowly sinuate-dentate. Inflorescence a spike. Flowers 4-merous, zygomorphic, opening near sunset; floral tube 4–14 mm; sepals 7–11(-14) mm; petals white, fading red, 6-10 mm; staminal filaments 4–8.5 mm, anthers 3-6 mm; style 12–26 mm. Capsule 7–13 × 3–5 mm, erect, the body ellipsoid or ovoid, 4-angled, distal half pyramidal, the base of the pyramidal portion distinctly bulging, then immediately and sharply constricted to the terete proximal part. Seeds (2-)3-4(-8), 2–2.5 × 1–1.25 mm, ovoid, usually flattened on one or several sides by crowding in the fruit, reddish brown. 2n = 28.

#### Phenology and distribution.

Flowering from May through July, but sporadically as late as November. *Oenothera hispida* grows in sandy loam soils from the eastern half of Texas south through Mexico as far south Oaxaca. It is naturalized in Arkansas (Sevier Co.), coastal southern California, Georgia (Glynn Co.); its current status in both Arkansas and Georgia should be verified. It is considered an invasive species in California.

**Figure 1.**
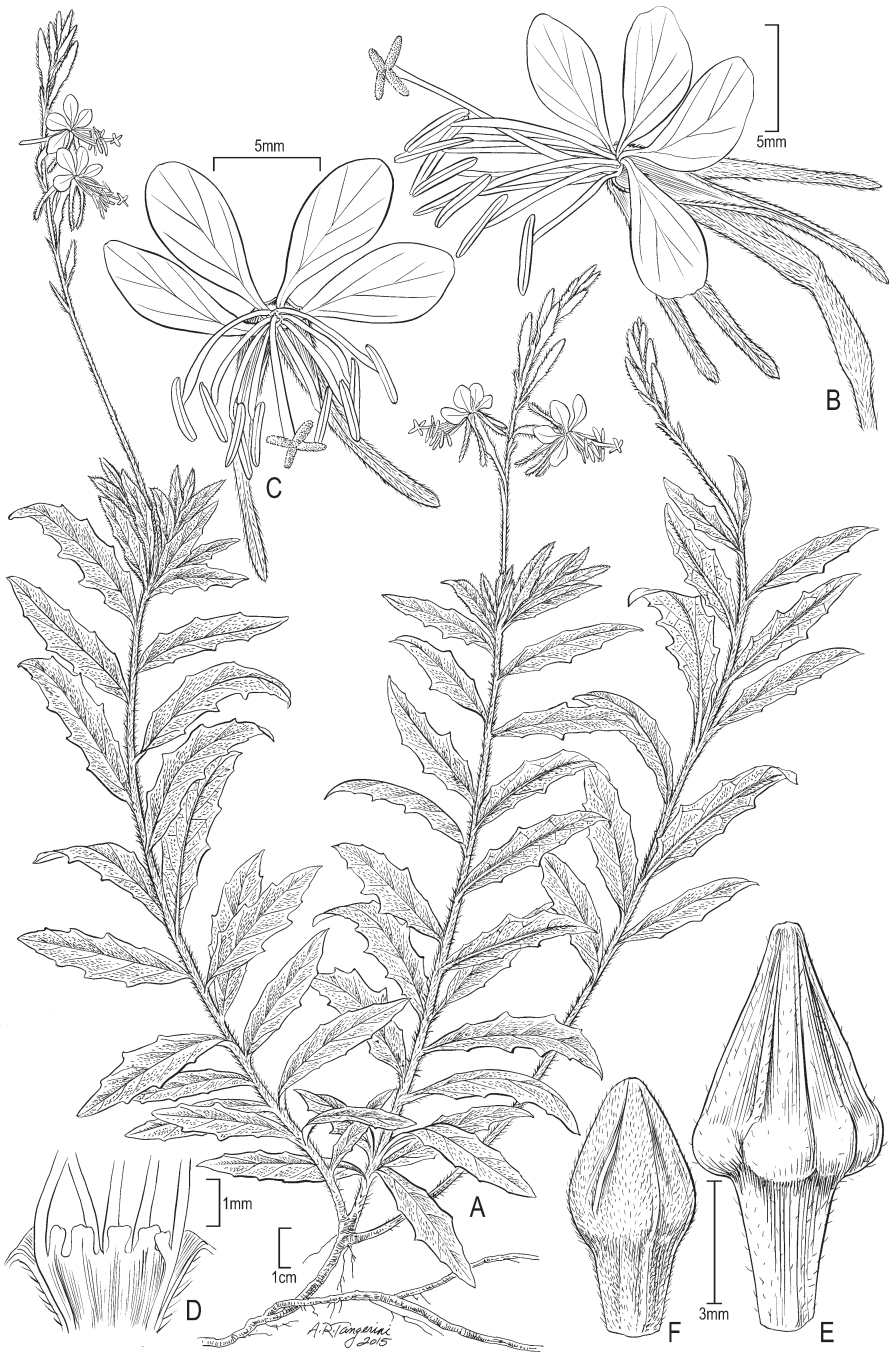
*Oenothera hispida* (Bentham) W.L.Wagner, Hoch & Zarucchi **A** Habit, Mexico, Nuevo León, *Roybal 34* (US) **B** Flower, lateral view, *Roybal 34* (US) **C** Flower, face view, digital image (Ray Pistrum as “Gaura drummondii fresh flower” [http://redsgoodvsevilcowbarn.blogspot.com/2012/06/chigger-chow-and-gaura-drummondii.html]) **D** Base of staminal filaments showing basal scales, *Roybal 34* (US) **E** Capsule, Texas, Hall 213 (US) **F** *Oenothera suffrutescens* (Ser.) W.L.Wagner & Hoch capsule, New Mexico, *Standley 6481* (US).

*Oenothera hispida* is the sole member of Oenothera sect. Gaura subsection Xenogaura. Raven and Gregory (1972) suggested that *Oenothera hispida* arose following interspecific hybridization between *Oenothera suffrutescens* (Ser.) W.L.Wagner & Hoch (subsect. *Campogaura* (P. H. Raven & D. P. Gregory) W.L.Wagner & Hoch) and a species in subsect. *Stipogaura* (P. H. Raven & D. P. Gregory) W.L.Wagner & Hoch, possibly near *Oenothera mckelveyae* (Munz) W.L.Wagner & Hoch. Hoggard et al. (2004) found that the pistillate parent of *Oenothera hispida* was indeed *Oenothera mckelveyae* or a close relative, but that the staminate parent probably came from a lineage related to *Oenothera dodgeniana* Krakos & W.L.Wagner or *Oenothera lindheimeri* (Engelm. & A.Gray) W.L.Wagner & Hoch in subsect. *Gaura* (L.) W.L.Wagner & Hoch. *Oenothera hispida* is not easily distinguished morphologically from *Oenothera suffrutescens* (subsect. *Campogaura*), with which it shares the character of a thick stipe, and occasionally hydridizes in Texas. *Oenothera hispida* is an aggressively rhizomatous perennial with fruits conspicuously bulging on the distal half (Raven and Gregory 1972). Since *Oenothera hispida* and *Oenothera suffrutescens* can be difficult to distinguish we have included a capsule of the latter in the figure (Fig. 1-F) for comparison of key features for correct separation of the two species. The rhizomatous habit makes this species potentially invasive, despite its self-incompatibility, but so far it has established itself most aggressively only in coastal southern California (Wagner et al. 2007). There are no other Hartweg collections of this species that anyone has seen other than the one cited above as the type collection. We have seen the holotype as an image on the Kew web site that was mistakenly filed under *Gaura coccinea* Pursh. The label information corresponds to the published locality given and is marked as in the Bentham herbarium.

## Supplementary Material

XML Treatment for Oenothera hispida (Bentham)

## References

[B1] Dietrich W (1978 [“1977”]) The South American species of Oenothera sect. Oenothera (*Raimannia*, *Renneria*; Onagraceae). Ann. Missouri Bot. Gard. 64: 425–626. doi: 10.2307/2395257

[B2] Ford VS Gottlieb LD (2007) Tribal relationships within Onagraceae inferred from PgiC sequences. Syst. Bot. 32: 348–356. doi: 10.1600/036364407781179725

[B3] Hoggard GD Kores PJ Molvray M Hoggard RK0 (2004) The phylogeny of *Gaura* (Onagraceae) based on ITS, ETS and *TrnL-F* sequence data. Amer. J. Bot. 91: 139–148. doi: 10.3732/ajb.91.1.139 21653370 10.3732/ajb.91.1.139

[B4] Levin RA Wagner WL Hoch PC Hahn WJ Rodriguez A Baum DA Katinas L Zimmer EA Sytsma KJ (2004) Paraphyly in Tribe Onagreae: Insights into phylogenetic relationships of Onagraceae based on nuclear and chloroplast sequence data. Syst. Bot. 29: 147–164. doi: 10.1600/036364404772974293

[B5] McNeill J Barrie FR Buck WR Demoulin V Greuter W Hawksworth DL Herendeen PS Knapp S Marhold K Prado J Prud’homme van Reine WF Smith GF Wiersema JH Turland NJ (Eds) (2012) International Code of Nomenclature for algae, fungi, and plants (Melbourne Code) adopted by the Eighteenth International Botanical Congress Melbourne, Australia, July 2011. Koeltz Scientific Books, Königstein, Germany [Regnum Veg. 154]

[B6] Raven PH Gregory DP (1972) A revision of the genus *Gaura* (Onagraceae). Mem. Torrey Bot. Club 23: 1–96.

[B7] Wagner WL Hoch PC Raven PH (2007) Revised classification of the Onagraceae. Syst. Bot. Monogr. 83: 1–240.

